# Editorial commentary on the special issue of immunology

**DOI:** 10.7555/JBR.40.20268001

**Published:** 2026-03

**Authors:** Editorial Board

Current immunological research covers a broad spectrum of areas, including host defense mechanisms, inflammatory signaling pathways, immune regulation, immunometabolism, and therapeutic interventions, with an increasing focus on systems immunology and multi-omics, next-generation cell therapies and biologics, and disease microenvironment analyses. This special issue comprises one review, eight original articles, and a letter covering diverse topics, including opioid-mediated immunomodulation, wound healing, osteoarthritis pathogenesis, neutrophil extracellular trap formation, vector control strategies, the genetic basis of the gut-skin axis, hepatitis C vaccine development, single-cell immune profiling in viral infection, neutralizing antibody discovery, and the association between vitamin D levels and cancer risk in psoriasis patients.

Gusakova *et al*^[1]^ reviewed the role of opioids in regulating immune cell function and reducing inflammatory cardiac injury. The authors summarized how opioid receptors, MRGPRX2, and TLR4 mediate the effects of opioids on neutrophils, macrophages, and T cells, and discussed the dual role of opioids in modulating proinflammatory cytokine production and reactive oxygen species generation, offering new insights into the immunomodulatory effects of opioid receptor activation in the context of cardiac reperfusion injury.

Zong *et al*^[2]^ applied remote neuromuscular electrical stimulation to skeletal muscle in a mouse wound model and demonstrated that it upregulated midkine expression through the AMPK-ERK axis, which in turn enhanced macrophage efferocytosis *via* LRP1 and accelerated wound healing. This study provides a mechanistic basis for remote neuromuscular electrical stimulation as a non-invasive therapeutic strategy for chronic wounds.

Shen *et al*^[3]^ investigated the effects of prolonged mechanical loading on osteoarthritis progression using both *in vitro* and *in vivo* models, and found that sustained moderate mechanical stress induced chondrocyte apoptosis and created a pro-inflammatory microenvironment mediated by nerve growth factor. These findings suggest that limiting high-impact or prolonged moderate-intensity exercise may benefit patients with early-stage osteoarthritis.

Grigorieva *et al*^[4]^ identified hypochlorous acid-modified human serum albumin as a novel inducer of reactive oxygen species-dependent suicidal NETosis involving myeloperoxidase, NADPH oxidase, and phosphatidylinositol 3-kinase signaling and demonstrated that a monoclonal antibody against hypochlorous acid-modified human serum albumin effectively blocked this effect, suggesting a potential therapeutic strategy for NETosis-related inflammatory diseases.

Zhao *et al*^[5]^ generated polyclonal antibodies against *Culex pipiens* antigens and fed them to mosquitoes, demonstrating that these antibodies impaired oviposition and increased mortality through classical complement pathway activation, with metabolic proteins identified as key targets, providing a proof-of-concept for developing transmission-blocking vaccines against mosquito-borne diseases.

Peng *et al*^[6]^ performed a genome-wide pleiotropic analysis using GWAS summary statistics and discovered extensive genetic correlations and 298 shared loci between skin and gastrointestinal tract diseases, with enrichment in immune-related pathways such as JAK-STAT and HLA-mediated immunity, supporting the concept of a gut-skin axis mediated by shared genetic factors.

Kuprianov *et al*^[7]^ engineered self-assembling peptide nanoparticles displaying the conserved HLA-A2-restricted hepatitis C virus NS3–1073 epitope and demonstrated that these nanoparticles activated human dendritic cells and induced NS3-specific T cell proliferation in mice, highlighting the potential of this platform for therapeutic vaccine development against hepatitis C.

Zhao *et al*^[8]^ performed single-cell RNA sequencing on peripheral blood mononuclear cells from patients with severe fever with thrombocytopenia syndrome (SFTS) and revealed that glucocorticoid treatment suppressed inflammation and interferon responses but inhibited virus-specific antibody production, elucidating the immunopathology of SFTS and the immunomodulatory effects of glucocorticoids.

Lu *et al*^[9]^ developed an epitope-guided negative screening strategy using phage display with a mutant receptor-binding domain and isolated six neutralizing antibodies against SARS-CoV-2 that exhibited broad-spectrum activity against viral variants and synergistic effects in combination, underscoring the efficiency of this screening approach.

Deng *et al*^[10]^ conducted a cross-sectional study using National Health and Nutrition Examination Survey (NHANES) data, and found that higher serum 25(OH)D levels, particularly above 100 nmol/L, were positively associated with cancer risk in adults with psoriasis, challenging the conventional protective view of vitamin D and highlighting the need for cautious interpretation and individualized monitoring of vitamin D supplementation in psoriasis patients.

We hope that the readers find this special issue insightful and inspiring.
